# Simple and inexpensive microwave setup for industrial based applications: Quantification of flower honey adulteration as a case study

**DOI:** 10.1038/s41598-024-59346-3

**Published:** 2024-04-17

**Authors:** Ugur C. Hasar, Hafize Hasar, Hamdullah Ozturk, Huseyin Korkmaz, Yunus Kaya, Mehmet Akif Ozkaya, Amir Ebrahimi, Joaquim J. Barroso, Vahid Nayyeri, Omar M. Ramahi

**Affiliations:** 1 Department of Electrical and Electronics Engineering, Gaziantep University, 27310 Gaziantep, Turkey; 2 Ministry of Agriculture and Forestry of Republic of Türkiye, Gaziantep Directorate of Provincial Agriculture and Forestry, 27090 Gaziantep, Turkey; 3 Department of Electrical and Electronics Engineering, Gaziantep Islam Science and Technology University, 27010 Gaziantep, Turkey; 4 Department of Electronics and Automation, Bayburt University, 69000 Bayburt, Turkey; 5 The School of Engineering, RMIT University, Melbourne, VIC Australia; 6 Instituto Tecnológico de Aeronáutica, São José dos Campos, SP 12228-900 Brazil; 7 School of Advanced Technologies, Iran University of Science and Technology, Tehran, 1684613114 Iran; 8 Department of Electrical and Computer Engineering, University of Waterloo, Waterloo, ON N2L 3G1 Canada

**Keywords:** Adulteration, Cheaper, Honey, Instrument, Microwave, Simple calibration, Water, Electrical and electronic engineering, Techniques and instrumentation, Engineering

## Abstract

A simple and inexpensive microwave measurement setup based on measurements of magnitudes of transmission properties ($|S_{21}|_{\text {dB}}$) is proposed for industrial-based microwave aquametry (moisture or water content) applications. An easy-to-apply calibration procedure based on normalization is implemented to eliminate systematic errors in the measurement system. As a case study, we applied this setup for the quantification of water-adulteration in flower honey. After validating this system by distilled water and pure flower honey measurements, $|S_{21}|_{\text {dB}}$ measurements of the pure flower honey with various adulteration percentages ($\delta$) up to 9% are conducted to examine the performance of the measurement setup for quantification of water adulteration. A multi-dimensional fitting procedure is implemented to predict $\delta$ using the proposed inexpensive microwave measurement setup. It is shown that it is possible to quantify an adulteration level with an accuracy better than ${\mp } 1$% by the proposed measurement setup and the applied multi-dimensional fitting procedure.

## Introduction

Electromagnetic spectrum covers various ranges of frequency bands including radio signals, microwaves, millimeter waves, terahertz, infrared radiation, visible light, ultraviolet radiation, X-rays, and gamma rays. Among these ranges, microwave frequencies, roughly from 300 MHz to 30 GHz, come forward especially for aquametry (moisture or water content) applications as reported in the books^1–3^, because water has dispersion characteristics or resonance behavior within microwave frequency band as noted in the study^4^. Conduction, dipolar, magnetization, resonance, and relaxation properties of a substance can be accounted by intrinsic electric permittivity and magnetic permeability properties^5^. Material characterization by way of these electromagnetic properties through microwave measurements has further gained importance in recent years in diverse applications including liquid detection^6,7^, damage detection of carbon-fiber reinforced composites^8^, determination of moisture content of grain products^9^, and breast cancer detection^10^. Microwave measurement techniques can be categorized into resonant and non-resonant techniques^11^. While resonant methods are highly accurate and sensitive^12,13^, they are mainly limited to material characterization measurement of low-loss materials because resonant frequency measurements are affected by high-loss materials due to broadening of the frequency spectrum. Another drawback of these methods is their bandwidth limitation (critical in characterizing dispersive materials)^14^.

Transmission line techniques, free-space techniques, and open-ended waveguide/coaxial probe techniques are examples of non-resonant microwave methods. Among these methods, free-space methods are non-contact^15–18^ and thus applicable for measurements of hazardous liquids or samples at high-temperatures^15^. Although accurate and non-contact, free-space measurements, in general, require samples with a large transverse area and application of microwave absorbers to eliminate the effects of diffraction at the samples’ edges.^18^. These effects can also be removed by using spot-focusing antennas^15–17^ which in turn limit the frequency band and increase overall budget of the measurement system especially for high-frequency measurements. Another issue related to free-space measurements is the effects of undesired signals from ground or between the antenna and the sample, which can overall affect the accuracy of the measurements. Time-domain gating can be used to eliminate such errors; however, it requires some preliminary information about the electromagnetic characteristics of the sample (the gating may require prior knowledge of the dispersive behavior of the sample) and thus is operator-dependent. Additionally, it also introduces some undesired frequency responses (unexpected ripples due to sharp frequency termination at the corners) around minimum and/or maximum frequencies in the band as noted in the study^19^. Open-ended waveguide aperture/coaxial probe measurements utilized in the studies^20–23^ can be used to examine near-field response of samples (solid, liquid or powder). However, these methods assume, in general, that the liquid/powder sample has large attenuation at the probe opening or the solid sample has a large surface area. Such a requirement might be problematic especially for low-loss samples with a relatively smaller size. In addition, any offset between the probe or aperture and the sample front surface^24^ can significantly affect the accuracy of measurements if care is not exercised or if the theory is not specialized for it. Transmission-line techniques, on the other hand, provide broadband frequency response of reflection and transmission properties of solid, liquid or powder samples. They are applicable for low-to-high-loss samples and require a lower budget compared to free-space methods as mentioned in the study^14^. As discussed in the studies^11,25,26^. Non-resonant microwave techniques have a relatively high accuracy and are broadband, which is especially useful for the characterization of dispersive materials. Furthermore, these methods require relatively less labor in the preparation or fabrication of samples.

Non-resonance transmission line techniques generally require complex scattering scattering (S-) parameter measurements. These measurements can only be performed by using expensive vector network analyzers (VNAs) as done in the studies^15–17,19,21,25–28^, which are extremely expensive. To reduce the overall cost of microwave measurement setups, especially for industrial applications, equipments operating based on amplitude-only measurements such as power sensor, amplitude analyzer, or crystal diode detector could be applied as implemented in the studies^14,29–39^. Because the measurement setups in the studies^29–35^ used reflected signals with or without transmission signals) in the analysis, directional couplers were needed to perform such measurements. On the other hand, the measurement setup used in the studies^36–39^ was based on transmission-only measurements so that a simple power meter could be implemented to pick up relevant transmitted signals through the sample. Therefore, the cost of the measurement setup in the studies^14,36–39^ is relatively lower than the cost of the measurement setups in the studies^29–35^. The method presented in the study^36^ is limited to characterization of high-loss samples (producing at least 10 dB attenuation). Besides, the methods in the studies^14,37–39^ can be applied for characterization of low-to-high loss samples.

In this study, as our first contribution, we propose another simple and relatively inexpensive microwave measurement setup for characterization of low-to-high loss materials based on transmission-only measurements. Our setup simply uses an oscillator, an adapter, an attenuator (depending on the need), a measurement cell, a diode-detector, and an analog dB meter. Therefore, the proposed setup is as relatively inexpensive as the setups in the studies^37–39,39^, which use a calibrated power sensor for transmission power detection in charge of detector and dB meter.

Produced by *Apis mellifera* bees mainly from nectar of flowers (fructose and glucose) and from secretions of plants, honey is considered to be one of the richest sources of sugar and thus has a key role in pharmaceuticals as a flavor of medications. In return, pure honey is relatively expensive and can be a subject of adulteration. Identifying pure honey from adulterated ones could be difficult for consumers by a simple color, concentration, and viscosity analysis as noted in the study^40^. To overcome this challenge, various techniques are utilized for detecting and quantifying the adulteration in pure honey such as liquid chromatography^41^, near-infrared spectroscopy^42^, mass spectrometry^43^, and microwave measurements (or sensors)^44^. Although liquid chromatography, near-infrared spectroscopy, and mass spectrometry are highly accurate, they are relatively expensive and need to be conducted by highly trained technicians. Therefore, there is a need for a fast, simple, and relatively inexpensive sensor for detection and quantification of adulteration in pure honey as referenced in the studies^40,45^. Microwave measurements are good candidates especially for such adulteration analysis because they are label-free, non-invasive, and provide instantaneous real-time response as noted in the study^46^.

Microwave techniques applied for adulteration detection within pure honey could be categorized into resonant methods^45^ and non-resonant methods^20,21,27,28,40,47,48^. Microwave measurements performed in these studies were implemented by using expensive VNAs. On the contrary, here, we apply the proposed measurement setup, as our second contribution, for detection and quantification of water-adulteration (or aquametry measurements as mentioned before) within pure flower honey samples as case study. Finally, different from earlier works in the studies^27,28^, which used one-dimensional fitting procedures, here, we apply a multi-dimensional fitting process, as our third contribution in the present study, for improving the accuracy in quantification of water adulteration within flower honey samples.

## Methodology

**Figure 1:**
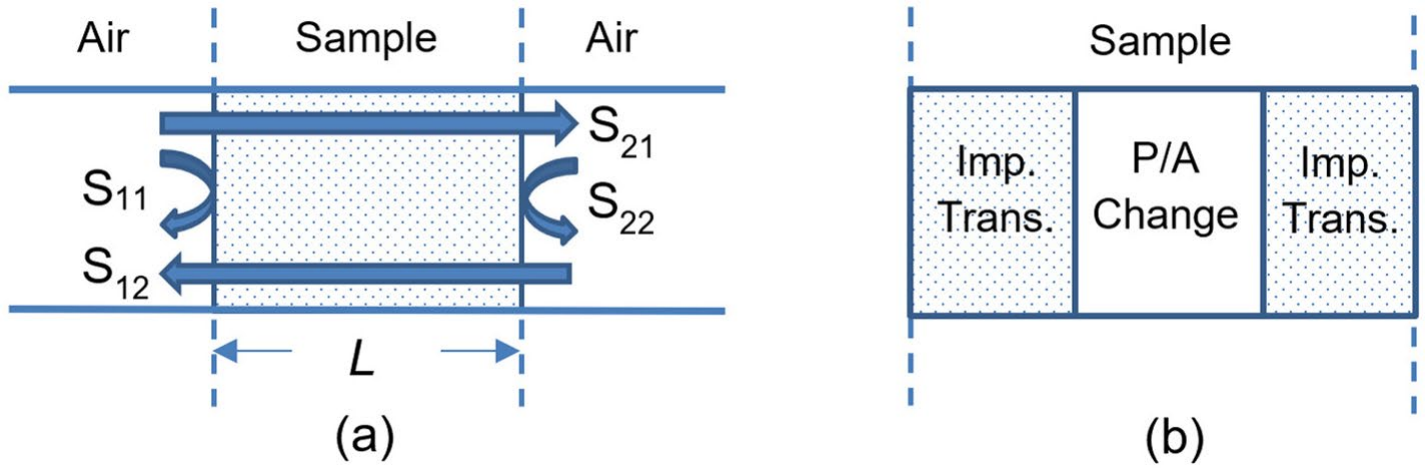
(**a**) Configuration of sample-filled waveguide section and (**b**) representation of the sample-filled waveguide in terms of impedance transition and amplitude and/or phase change^49^.

Figure 1a illustrates a dielectric sample (honey samples in general possess dielectric property only) with a relative complex permittivity $\varepsilon _r$ and length *L* loaded into a rectangular metallic hollow waveguide operated in its dominant mode TE_10_. The wave-cascading matrix of the sample [*M*] in reference to empty (air) waveguide sections can be written as given in the study^49^

1 $$\begin{aligned}{}[M] = \begin{bmatrix} 1 &{} \Gamma \\ \Gamma &{} 1 \\ \end{bmatrix} \begin{bmatrix} T &{} 0 \\ 0 &{} 1/T \\ \end{bmatrix} \begin{bmatrix} 1 &{} \Gamma \\ \Gamma &{} 1 \\ \end{bmatrix}^{-1}, \end{aligned}$$

where

2 $$\begin{aligned} \Gamma = \dfrac{Z - Z_0}{Z - Z_0}, \ \ \ T = e^{- \gamma L}, \ \ \varepsilon _r = \varepsilon _r^{\prime } - j \varepsilon _r^{\prime \prime }, \end{aligned}$$

3 $$\begin{aligned} Z = j \omega \mu _0 / \gamma , \ \ Z_0 = j \omega \mu _0 / \gamma _0, \end{aligned}$$

4 $$\begin{aligned} \gamma = j k_0 \sqrt{ \varepsilon _r - \left( f_c/f \right) ^2}, \ \ \gamma _0 = j k_0 \sqrt{ 1 - \left( f_c/f \right) ^2}. \end{aligned}$$

here $\Gamma$ is the reflection coefficient at the air-sample interface, and *T* is the propagation factor within the sample. *Z* and $Z_0$ are the wave impedances of the sample-filled and air-filled waveguide sections. $\gamma$ and $\gamma _0$ are the propagation constants of the sample-filled and air-filled waveguide sections. $\mu _0$ is the permeability of the free-space, $\omega$ is the angular frequency, $f_c$ and *f* are the cutoff and operating frequencies, and $\varepsilon _r^{\prime }$ and $\varepsilon _r^{\prime \prime }$ are the real and imaginary parts of $\varepsilon _r$, which indicates the degree to which a material can be polarized and dissipation within a dielectric sample, respectively.

As shown in Fig. 1b, the first and third matrices in Eq. (1) denote impedance transitions while the middle one corresponds to the amplitude and/or phase change. The matrix [*M*] is a function of the measured S-parameters as implemented in the study^49^

5 $$\begin{aligned}{}[M] = \frac{1}{S_{21}} \begin{bmatrix} ( S_{12} S_{21} - S_{11} S_{22} ) &{} S_{11} \\ -S_{22} &{} 1 \\ \end{bmatrix}, \end{aligned}$$

where $S_{11}$, $S_{22}$, $S_{21}$, and $S_{12}$ are the forward and backward reflection and transmission S-parameters.

From Eqs. (1) and (5), it is possible to write $S_{21}$ as shown in the studies^14,36^

6 $$\begin{aligned} S_{21}= & {} \dfrac{ \Gamma (1 - T^2) }{1 - \Gamma ^2 T^2} \nonumber \\ {}= & {} \dfrac{4 \gamma \gamma _0 T }{ (\gamma + \gamma _0)^2 - (\gamma - \gamma _0)^2 T^2}. \end{aligned}$$

Our goal is to measure $|S_{21}|$ and detect and quantify the water adulteration in honey samples by means of $|S_{21}|$.

## Measurement setup

**Figure 2:**
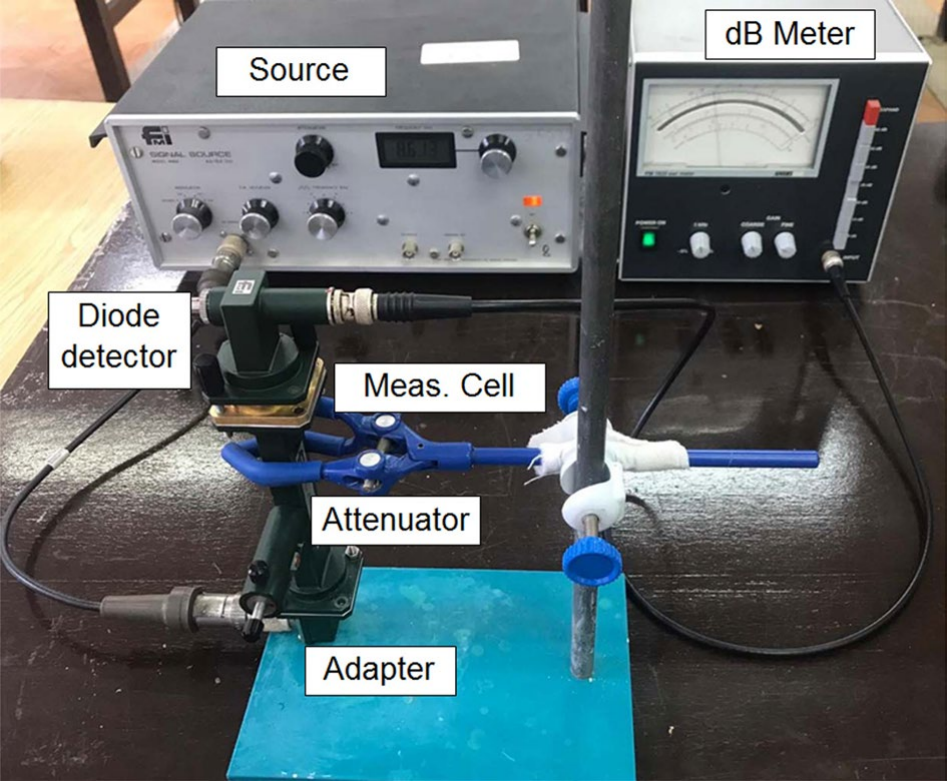
A picture of the microwave measurement setup.

A simple microwave measurement setup, as shown in Fig. 2, was constructed to measure $|S_{21}|$ of a waveguide section entirely loaded by flower honey (pure or adulterated). The setup was positioned vertically to conveniently perform $|S_{21}|$ measurements of the examined honey samples. The process of pouring or loading the sample into the measurement cell will be discussed in Subsections 3.1 and 3.2. This setup consists of simple microwave equipments including a microwave source, an adaptor, an attenuator, a waveguide measurement cell, a diode-detector, and an analog dB meter. The source from Flann Microwave Instruments (FMI) with model 449X operates at X-band (between 8.2 and 12.4 GHz) producing an output signal of approximately 10 mW. An adaptor from FMI with model 16093 operates as a guide to direct signals from the source to the attenuator and waveguide. The attenuator from FMI with model 16080 is used to protect the source from being overloaded by the reflected signals. A fraction of the source power after the attenuator is then transferred to the measurement cell, which houses the flower honey samples with different percentages of water adulteration. The diode-detector from FMI with model 16180, which works on the principle of square-law, at the end of the setup is used to pick up the transmitted signals $|S_{21}|$. Compared with the setups used in earlier works^20,21,27,28,40,45,47^, the measurement setup in Fig. 2 can be considered to be appreciably less expensive since it does not require any expensive VNA instrument and even any directional couplers as shown in the study^14^. Furthermore, the proposed setup is as relatively inexpensive as the setups in the studies^14,37–39^, which use a calibrated power sensor for transmission power detection in charge of detector and dB meter.

Because the setup is based on $|S_{21}|$ measurements, a suitable yet accurate calibration procedure should be implemented to eliminate some systematic errors in the measurement system. Since the measured signals are proportional to the square of the transmitted signals through the sample (i.e., the principle of square-law detection), and provided that the amplitude of the transmitted signal is above the noise level and is not too large, as detailed in the book^50^, we applied the calibration procedure in the study^36^

7 $$\begin{aligned} |S_{21}| = \sqrt{ \dfrac{ T_s (\omega ) - T_m (\omega ) }{ T_f (\omega ) - T_m (\omega )} }, \end{aligned}$$

where $T_s (\omega )$ is the measured transmitted signal when the measurement cell is loaded with pure or adulterated flower honey, $T_f (\omega )$ is the measured transmitted signal when the measurement cell is unloaded (no sample); and $T_m (\omega )$ is the measured transmitted signal when a metal plate is located at the end of the measurement cell. It is noted that $T_m (\omega )$ corresponds to noise signals when no energy is present and is included into Eq. (7) to improve the measurement accuracy.

## Results and discussion

All the measurements of distilled water, pure flower honey, and water-adulterated flower honey in this section were implemented at ordinary laboratory conditions (temperature: $23 {\mp } 1$ ^∘^C and relative humidity: 50–60%).

### Validation

**Figure 3:**
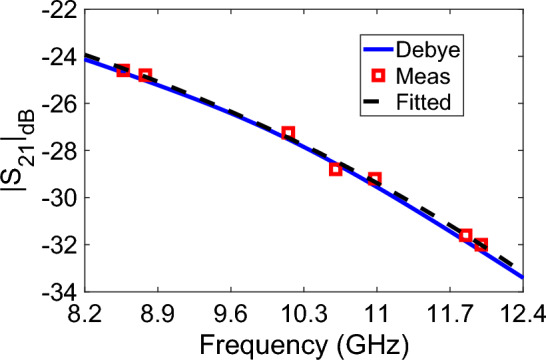
(Measured (shown by a red square symbol), calculated (shown by a blue solid line—Debye model), and fitted (shown by a black dashed line) $|S_{21}|_{\text {dB}}$ of the distilled water at X-band by the proposed setup.

Before carrying out water-adulteration analysis by the simple and cheaper microwave measurement setup in Fig. 2, we performed some preliminary measurements to validate the simple calibration process based on averaging in Eq. (7), and to examine the accuracy of the setup. For this goal, we measured $|S_{21}|$ (dB) (henceforth denoted as $|S_{21}|_{\text {dB}}$) of distilled water ($L \cong 5.0$ mm) poured in the opening of the cell with length $L_g = 10.16$ mm. Here, a thin adhesive tape was applied to cover the entire opening at the bottom of the cell. In this way, there is no need to use a dielectric plug/window to hold the liquid sample in place in the cell as exercised in the study^51^. Figure 3 illustrates the measured $|S_{21}|_{\text {dB}}$ of the distilled water (shown by a red square symbol) averaged from three independent measurements at some discrete frequencies at X-band. In order to compare the accuracy of measurements, calculated $|S_{21}|_{\text {dB}}$ values of the distilled water were also calculated by using the Debye model with one-pole as implemented in the studies^14,27,38^

8 $$\begin{aligned} \varepsilon _r = \varepsilon _{\infty } + \dfrac{ \varepsilon _s - \varepsilon _{\infty } }{ 1 + j \omega \tau }, \end{aligned}$$

where $\varepsilon _s$ and $\varepsilon _{\infty }$ are, respectively, the relative permittivity at considerably small (theoretically zero) and considerably high (theoretically infinite) frequencies, and $\tau$ is the relaxation time (rearrangement time). For distilled water, $\varepsilon _s = 78.5$, $\varepsilon _{\infty } = 5.2$, and $\tau = 8.33$ ps^52^. It is seen from Fig. 3 that the measured and calculated $|S_{21}|_{\text {dB}}$ values from Eqs. (2)–(6) are in good agreement at the discrete frequencies, with differences not exceeding 2%. These results partly show that the measurement setup has sufficient accuracy to quantify the water-adulteration level within flower honey samples.

In order to examine whether the measured $|S_{21}|$ values at discrete frequencies are in good agreement with the curve of the Debye model over the entire frequency band, we applied an exponential curve fitting for the measured $|S_{21}|_{\text {dB}}$ ($|S_{21}^f|_{\text {dB}}$) using the following expression

9 $$\begin{aligned} |S_{21}^f|_{\text {dB}} = a e^{b f_{\text {GHz}}} + c, \end{aligned}$$

where *a*, *b*, and *c* are the curve fitting parameters, and $f_{\text {GHz}}$ is the frequency in GHz. Utilizing the measured $|S_{21}|_{\text {dB}}$ at discrete frequencies in Fig. 3, the parameters *a*, *b*, *c* were determined as shown in Table 1. This table also presents the value of $R^2$ which means how well the measured data fit to the fitting expression in Eq. (9) ($R^2 = 1.0$ means the best fitting). It is noted from Fig. 3 that the measured data fitted by these parameters follow the data of the Debye model very closely.

**Table 1:** Curve fitting values *a*, *b*, and *c* using the measured $|S_{21}|$ at discrete frequencies, together with the $R^2$ value, for distilled water and pure flower honey.

Sample	Parameter			$R^2$
	*a*	*b*	*c*	
Distilled water	− 4.653	0.1271	− 10.61	0.985
Pure Honey	1650	− 0.8818	− 9.068	0.939

**Table 2:** Physicochemical properties (including standard maximum or minimum limits) of the tested flower honey along with measurement techniques.

Parameter	Value	Measurement methodology	Max or min value
Color	52 mm Pfund	Tintometer	–
Moisture	16.51%	Refractometer	Max. 20
Free acidity	19.70 meq/kg	Titrimetric	Max. 50
pH	3.80		–
Diastase number	9.8 DN	Spectrometer	Min. 8
Hydroxymethylfurfural (HFM) level	25.76 mg/kg		Max. 40
Proline	437 mg/kg		Min. 300
Conductivity	0.308 mS/cm	Conductivity meter	Max. 0.8
Fructose	37.67%		–
Glucose	31.13%	High-performance liquid chromatography	–
Sucrose	1.03%	(HPLC)	Max. 5
Maltose	1.79%		Max. 4
$\delta ^{13}$ C_Protein_	− 2.5210%	LC-isotope ratio mass spectrometry	–
$\delta ^{13}$ C_Honey_	− 2.5188		Max. − 2.3

### Sample preparation and environmental conditions

**Figure 4:**
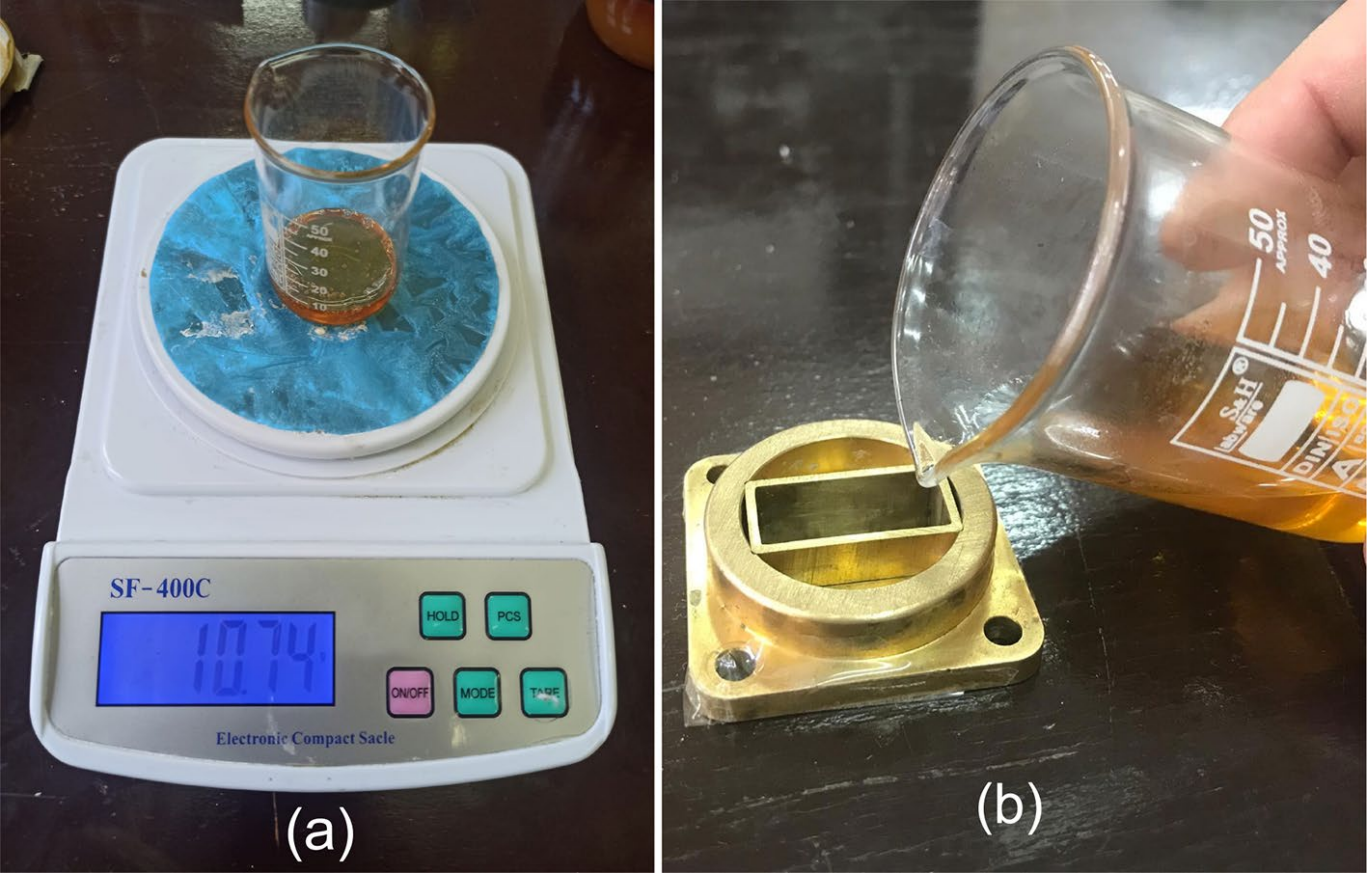
(**a**) Water-adulterated (9%) flower honey within a beaker positioned on the used scales and (**b**) sample pouring into the measurement cell.

Pure flower honey blended from different origins of Anatolia regions in Türkiye, purchased from a local market, was used to examine the efficiency of the proposed method. Details about the physicochemical properties (including standard maximum or minimum limits) of the tested flower honey along with measurement techniques are presented in Table 2. Tested flower honey was stored within its original glass container throughout all tests to minimize the effect of storage conditions on microwave measurements. Honey samples were prepared at ordinary room conditions (a temperature of ${\mp } 23^{\circ }$C and a relative humidity of approximately 55%). The water adulteration level was arranged on the mass-to-mass basis procedure by using precision scales (hodbehod SF-400C) with 0.01 g accuracy and 600 g maximum capacity. It is noted that distilled water and pure flower honey in proper amounts were mixed in a 50 mL glass beaker by a stirrer with a constant rotational speed of approximately 30 seconds to mitigate any formation of any air bubbles, especially for mixtures with higher water adulteration. Such a step is necessary because viscosity of distilled water is considerably lower than the viscosity of pure flower honey as discussed in the studies^20,21,27^. While adulteration levels of $\delta = 3$, 6, and 9 (each of which corresponds to the percentage of adulteration calculated by the mass-to-mass ratio of water to pure flower honey) were considered in the analysis of the fitting procedure, adulteration levels of $\delta = 4$, 5, 7, and 8 were utilized for testing the validity of the fitted formula used for the adulteration quantification procedure, as discussed in Subsection 3.4. We restricted our analysis to the values of $\delta$ less than 10% because any value of $\delta$ over or around 10 not only produces a considerable change in viscosity of pure flower honey but also results in a substantial difference in its color and taste^53^. For each adulteration level, three sample sets were prepared to minimize any possible measurement errors (a total of 24 samples including the pure flower honey). The masses of each pure or water-adulterated flower honey sample used in each experiment were set at approximately 10g. We paid attention to the determination of this mass value by considering that it would be relatively much so that its value measured by the used scales was sufficiently accurate. Figure 4a shows water-adulterated (9%) flower honey within a beaker on the used scales.

A thin and widely used adhesive tape was applied to fill the entire opening at the bottom of the cell. Then the prepared mixtures of adulterated flower honey samples were carefully poured into the opening (hollow waveguide) of the measurement cell, as shown in Fig. 4b. Because of the relatively high viscosity of the flower honey samples, special care was exercised in cleaning the measurement cell with acetone after the mixture was poured back into the beaker for future use and after the adhesive tape was removed. Thereafter, the measurement cell was left at ordinary room conditions for about one minute in order for the acetone to evaporate. Such a two-step cleaning procedure was observed to be effective in reducing the effect of drift of the first experiment to the last one for repeated measurements as implemented in our previous study^27^.

### Water-adulterated measurements

**Figure 5:**
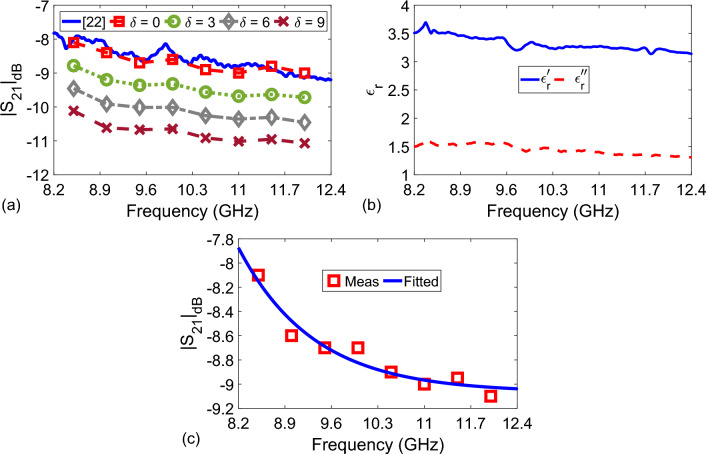
(**a**) Measured $|S_{21}|_{\text {dB}}$ at some discrete frequencies (8.5:0.5:12.0 GHz) for adulteration percentages of $\delta = 0$, 3, 6, and 9, (**b**) extracted $\varepsilon _r^{\prime }$ and $\varepsilon _r^{\prime \prime }$ of the pure flower honey by using the method discussed in the study^27^, and (c) measured $|S_{21}|_{\text {dB}}$ (Meas) of the pure flower and fitted $|S_{21}|_{\text {dB}}$ using *a*, *b*, and *c* values in Table 1 (Fitted).

After validating our measurement apparatus using transmission $|S_{21}|_{\text {dB}}$ measurements for the distilled water, we continued with $|S_{21}|_{\text {dB}}$ measurements of pure flower honey with and without water-adulteration. Presented results here and in the following subsection were obtained by averaging $|S_{21}|_{\text {dB}}$ from three sets for each $\delta$ level. Figure 5a illustrates the variation of $|S_{21}|_{\text {dB}}$ at some discrete frequencies (from 8.5 GHz to 12 GHz with 0.5 GHz increments) for different adulteration levels of $\delta = 0$, 3, 6, and 9. As expected, it is seen from Fig. 5a that $|S_{21}|_{\text {dB}}$ decreases with an increase in $\delta$, since water has a loss factor considerably greater than that of pure flower honey tested in our study, as validated by earlier works^27,28^.

In order to examine whether $|S_{21}|_{\text {dB}}$ for the tested pure flower honey ($\delta = 0$) is accurate, we also performed additional measurements. To this end, we applied the methodology in our recent study^27^ and extracted $\varepsilon _r$ of pure flower honey using a waveguide setup operating at X-band. Figure 5b gives the extracted $\varepsilon _r^{\prime }$ and $\varepsilon _r^{\prime \prime }$ of the tested pure flower honey over X-band. Next, we substituted $\varepsilon _r^{\prime }$ and $\varepsilon _r^{\prime \prime }$ values into Eqs. (2)–(6) to calculate $|S_{21}|_{\text {dB}}$, which is presented in Fig. 5a. It is seen from Fig. 5a that the measured $|S_{21}|_{\text {dB}}$ by the proposed measurement setup and calculated $|S_{21}|_{\text {dB}}$ after using the expensive setup in the study^27^ are in good agreement. To examine the fitness of the expression in Eq. (9) for pure flower honey, we first determined the fitting parameters *a*, *b*, and *c*, as presented in Table 1, and then drew the dependence of $|S_{21}|$ versus frequency, as shown in Fig. 5c. When the fitting parameters in Table 1 were utilized in Eq. (9), it was observed from Fig. 5c that the measured and fitted $|S_{21}|_{\text {dB}}$ for the pure flower honey are in good agreement ($R^2$ value is approximately 0.94).

### Multi-dimensional fitting process for evaluation of adulteration level

It is seen from Fig. 5a that for a given adulteration level $\delta$, one (*a*, *b*, *c*) combination could produce an optimum solution by using one-dimensional fitting process. However, as different from previous works^27,28^ both of which use one-dimensional fitting procedures, for a set of adulteration levels (e.g., $\delta = 0$, 3, 6, and 9), an optimum solution for one (*a*, *b*, *c*) set needs to be sought for by using a multi-dimensional fitting process. For this goal, as a first step, a possible function which could be used for describing the behavior of $|S_{21}|_{\text {dB}}$ needs to be defined. It was demonstrated by measurements in the study^27^ that variations in $\varepsilon _r^{\prime }$ and $\varepsilon _r^{\prime \prime }$ for a small increased adulteration ($\delta =$ 0:10) change can be related to a linear function of $\delta$. As can be seen from Fig. 5a that a similar relationship is also noted between $|S_{21}|$ and $\delta$. Therefore, we decided to use the following metric function for examining the variation of $|S_{21}|$ with $\delta$:

10 $$\begin{aligned} |S_{21}^f|_{\text {dB}} = - k \delta + a_{\text {all}} e^{b_{\text {all}} f_{\text {GHz}}} + c_{\text {all}}, \end{aligned}$$

where $a_{\text {all}}$, $b_{\text {all}}$, $c_{\text {all}}$, and *k* are the coefficients to be determined, which reflect an optimum (*a*, *b*, *c*) set for all adulteration levels of $\delta =$ 0, 3, 6, and 9.

**Figure 6:**
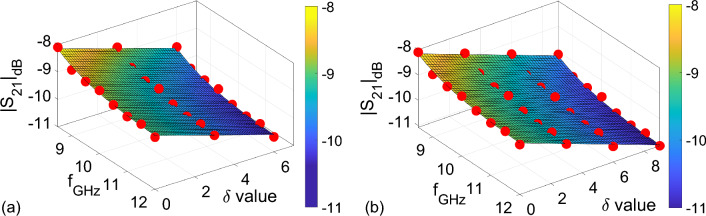
Dependencies of $|S_{21}|_{\text {dB}}$ versus $f_{\text {GHz}}$ and $\delta$ for (**a**) Case-I: $\delta = 0$, $\delta = 3$, and $\delta = 6$ and (**b**) Case-II: $\delta = 0$, $\delta = 3$, $\delta = 6$, and $\delta = 9$.

As a second step, we implemented the ‘fit’ function of MATLAB©for the analysis of multi-dimensional fitting process, with the fittype defined using Eq. (10) and non-linear least squares as the fit method. Figure 6a,b illustrate the fitting result obtained by using this function for two different cases as (a) Case-I: $\delta = 0$, $\delta = 3$, and $\delta = 6$ and (b) Case-II: $\delta = 0$, $\delta = 3$, $\delta = 6$, and $\delta = 9$. These two cases, as to be discussed shortly, were used to examine the effect of increasing the sampling number on the quantification of adulteration level. It is seen from Fig. 6a,b that the fitted 2D plane of $f_{\text {GHz}}$ and $\delta$ produces $|S_{21}|_{\text {dB}}$ values close to those of the measured ones presented by solid red circles. In order to quantitatively analyze the dependencies in Fig. 6a,b, $a_{\text {all}}$, $b_{\text {all}}$, $c_{\text {all}}$, and *k* were calculated for these two different cases, as presented in Table 3. It is seen from Table 3 that R-square value for the Case-II is greater than that of the Case-I. This implies that the optimum fitting could be implemented by using larger number of samples.

**Table 3:** Curve fitting values $a_{\text {all}}$, $b_{\text {all}}$, $c_{\text {all}}$, and *k* using the measured $|S_{21}|$ for different $\delta$ values.

Cases	Parameter				$R^2$
	$a_{\text {all}}$	$b_{\text {all}}$	$c_{\text {all}}$	*k*	
Case-I	667.5	0.7719	− 9.102	0.2234	0.926
Case-II	686.8	0.7763	− 9.099	0.2242	0.938

Case-I and Case-II refer, respectively, to the analysis performed for $\delta = 0$, 3, 6 and the analysis performed for $\delta = 0$, 3, 6, and 9.

**Figure 7:**
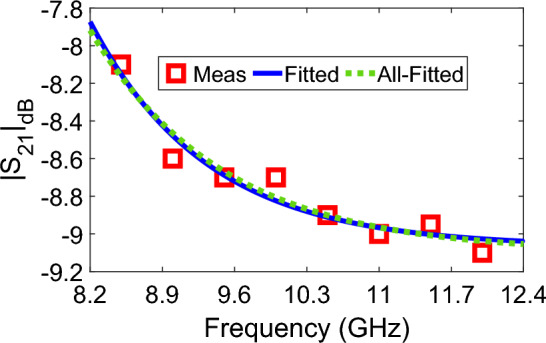
Measured $|S_{21}|_{\text {dB}}$ (Meas) of the pure flower, fitted $|S_{21}|_{\text {dB}}$ using *a*, *b*, and *c* values in Table 1 (Fitted), and fitted $|S_{21}|_{\text {dB}}$ using $a_{\text {all}}$, $b_{\text {all}}$, and $c_{\text {all}}$ values of the Case-II in Table 3 (All-Fitted).

It is instructive to examine whether $a_{\text {all}}$, $b_{\text {all}}$, $c_{\text {all}}$, and *k* values for Case-II (Table 3) for $\delta = 0$ produce results similar to those calculated by using *a*, *b*, and *c* values (Table 1). For this goal, we obtained $|S_{21}|_{\text {dB}}$ values for the pure flower honey ($\delta = 0$) shown in Fig. 7. It is seen from Fig. 7 that $|S_{21}|_{\text {dB}}$ values calculated by $a_{\text {all}}$, $b_{\text {all}}$, $c_{\text {all}}$, and *k* values for the Case-II (Table 3) and by using *a*, *b*, and *c* values (Table 1) are in good agreement.

**Figure 8:**
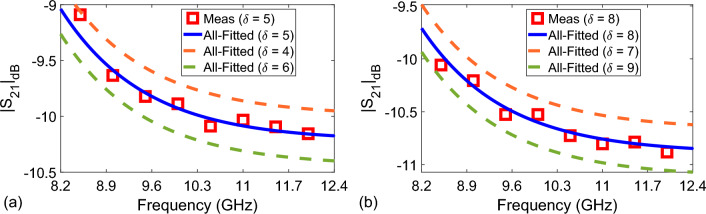
Measured and fitted $|S_{21}|_{\text {dB}}$ values (using $a_{\text {all}}$, $b_{\text {all}}$, and $c_{\text {all}}$ values of the Case-II in Table 3 (All-Fitted)) of the flower honey with (**a**) $\delta = 5$ (${\mp } 1$) and (**b**) $\delta = 8$ (${\mp } 1$).

**Table 4:** Comparison of our study with studies on microwave quantification/detection of honey adulteration in the literature.

Metric	Studies			
	^45^	^20,21,27,28,47^	^40,48^	This work
Meas. type	Resonant	Non-resonant		
Analysis type	Detection & quantification		Detection only	Detection & quantification
Overall Cost	Expensive requiring VNA instrument			Inexpensive

Finally, to evaluate the performance of the proposed relatively inexpensive microwave measurement setup and multi-dimensional fitting process through the expected functional behavior in Eq. (10), we performed $|S_{21}|_{\text {dB}}$ measurements of the tested flower honey with $\delta = 5$ and $\delta = 8$. Figure 8a,b demonstrate the measured $|S_{21}|_{\text {dB}}$ of the tested flower honey with $\delta = 5$ and $\delta = 8$ because they were not taken into account in the curve fitting analysis. In addition, these figures show fitted $|S_{21}|_{\text {dB}}$ values using $a_{\text {all}}$, $b_{\text {all}}$, and $c_{\text {all}}$ values of the Case-II in Table 3 (All-Fitted) for $\delta = 4$, 6, 7, and 9. It is noted from Fig. 8a,b that the measured $|S_{21}|_{\text {dB}}$ with $\delta = 5$ and $\delta = 8$ are, respectively, lying within $4< \delta < 6$ and $7< \delta < 9$ adulteration ranges predicted by the $a_{\text {all}}$, $b_{\text {all}}$, and $c_{\text {all}}$ values. This indicates that our proposed measurement setup, along with the fitting model, can predict accurate $\delta$ values within ${\mp } 1$% $\delta$ range for adulteration levels up to $\delta = 10$. This means that by drawing a grid of spectral $|S_{21}^f|_{\text {dB}}$ dependencies, each of which corresponds to different adulteration levels from $\delta = 1$ to $\delta = 10$ with 1% increment using Eq. (10), it is possible to predict water adulteration level within ${\mp } 1$% $\delta$ range.

### On the fitting procedure and applicability of our measurement setup

It should be emphasized here that in our analysis the fitting process implemented and the expression used in the fitting process may not be the best choice but were selected with the sole purpose of demonstrating the applicability of the proposed simple and inexpensive measurement setup. If needed, other fitting processes and fitting expressions can be used to improve the accuracy of quantification. Besides, it is noted that our proposed microwave measurement setup was validated, as a case study, by only one type of honey (flower honey). Various honey types different than flower honey such as highland and thyme honey are available, and yet physicochemical properties of even one certain type of honey can change due to its location and origin. Nonetheless, previous works in the literature^20,21,27,28,40,45,47,48^ and sensitivity of microwave signals to a change in water content inside various samples (aquametry) could be partially and potentially considered as concrete bases for our microwave setup to be actively used for quantification of water-adulteration of various honey samples (detection of minimum $\delta$ may possibly change from honey type and origin). It is also important to discuss that different from or in addition to the validation of our measurement setup by distilled water measurements (before its application to the quantification of water adulteration of flower honey), the Karl Fischer titration method^54^ could be potentially and equally used for evaluation of our measurement setup for quantification of water adulteration of flower honey. However, such a measurement technique requires expensive apparatus currently not available in our laboratory. On the other hand, the implemented multi-dimensional fitting process is limited to X-band measurements for the tested honey type only. If measurements were performed at another waveguide frequency band such as 3.95–5.85 GHz (WR187) for the same honey type or if measurements were conducted at the same band for a different honey type, then the proposed multi-dimensional fitting process must be re-implemented, which is considered as a disadvantage of our proposed technique.

Besides, it is instructive to make a comparison of our study with studies on microwave quantification/detection of honey adulteration in the literature. Table 4 illustrates such a comparison of our work with those studies in the literature^20,21,27,28,40,45,47,48^ in terms of measurement type, overall cost, and analysis type. It is noted from Table 4 that while the method in the study^45^ is a resonant method, methods in those studies^20,21,27,28,40,47,48^ and our work are non-resonant methods. Besides, the studies^40,48^ focus on detection of adulteration in honey only whereas the studies^20,21,27,28,45,47^ and our work concentrate not only on detection but also on quantification of adulteration in honey. Finally, while the measurement setups in the studies^20,21,27,28,40,45,47,48^ use expensive VNA instruments, the setup is in the present study is relatively inexpensive without requiring any VNA instrument. The disadvantage of our measurement setup, however, is that it can perform $|S_{21}|$ measurements at discrete frequencies only.

Finally, the proposed microwave sensor based on waveguide measurements, which is applied for quantification of water-adulteration in flower honey as a case study, can find applications for low-lost microwave aquametry applications of granular or liquid samples such as moisture detection of grains, soil samples, and food products^55^.

## Conclusion

A simple and relatively inexpensive microwave measurement setup is introduced for industrial-based applications especially for microwave aquametry applications. The setup uses only $|S_{21}|_{\text {dB}}$ measurements, which could be realized by a typical source, an adapter, an attenuator, a waveguide measurement cell, a diode-detector, and an analog dB meter. As a case study, this setup was tested for the quantification of water-adulteration of pure flower honey. Systematic errors in the measurement system were removed by an easy-to-apply calibration procedure based on normalization. In the quantification process, because the one-dimensional fitting procedure is limited for our analysis, a multi-dimensional fitting procedure based on the ‘fit’ function of MATLAB© along with the metric function in Eq. (10) involving an exponential decay with some offset is applied for evaluating the performance of the proposed measurement system. It is observed that the proposed measurement system and the implemented fitting procedure allow accurate quantification of water-adulteration of the tested pure flower honey up to ${\mp } 1$% within an adulteration limit of 10%.

## Data Availability

The datasets used and analyzed during the current study are available from the corresponding author (U.C.H) upon reasonable request.
